# Prediction of Crack Resistance of LFSMA-13 with and without Anti-Rut Agent Using Parameters of FTIR Spectrum under Different Aging Degrees

**DOI:** 10.3390/ma14123209

**Published:** 2021-06-10

**Authors:** Xing Wu, Aihong Kang, Bangwei Wu, Keke Lou, Zhao Fan

**Affiliations:** 1 College of Civil Science and Engineering, Yangzhou University, Yangzhou 225100, China; wuxingjs@gmail.com (X.W.); wubw@yzu.edu.cn (B.W.); lkkyzu@163.com (K.L.); 2 Research Center for Basalt Fiber Composite Construction Materials, Yangzhou 225127, China; 3 Jiangsu Expressway Company Limited, Nanjing 210000, China; f18752781240@126.com

**Keywords:** anti-rut agent, cracking resistance, FTIR spectrum, grey correlation, prediction model

## Abstract

This paper aims to better analyze the crack resistance of lignin fiber reinforced SMA-13 (LFSMA-13) asphalt mixtures, with and without polymer anti-rut agent (ARA), under different aging degrees. IDEAL-CT test and Fourier transform infrared (FTIR) spectroscopy were utilized to analyze the relationships between the crack resistance of LFSMA-13, with and without ARA, and the parameters of the FTIR spectrum of the asphalt extracted from the test samples. A convenient testing method to predict the anti-crack ability of the mixtures in a road was also derived in this study. The test samples were prepared using the specifications listed by AASHTO. The fracture formation work (W_initial_) and cracking index (CT_Index_) in the IDEAL-CT test were adopted to reflect the cracking ability of the asphalt mixtures in both the crack formation stage and the crack propagation stage. The peak areas of the FTIR spectrum were utilized to reveal the chemical properties of the asphalt material inside the SMA-13 asphalt mixtures, with and without ARA under different aging degrees. Grey correlation analysis was adopted to choose the most suitable FTIR spectrum parameters to derive the prediction models of W_initial_ and CT_Index_ under different aging degrees. After conducting a series of tests, the results showed that the aging process could well affect the crack resistance of the test samples and the peak areas of the asphalt extracted from the mixtures. The FTIR parameters selected from the grey correlation analysis could be used to well predict the anti-crack ability of the asphalt mixtures.

## 1. Introduction

Nowadays, pavements are facing more and more distress due to the fact that the increasing number of vehicles or loads on roads cause asphalt mixtures bear more inner stress, which causes the tendency towards experiencing distress [1,2,3], especially when mixtures are put through the process of aging [4,5,6] after being put into service. Micro cracks are usually seen as the initial distress because they can often result in stress concentration phenomenon [7,8,9] in pavement structures, which can cause more serious problems, such as bigger cracks [10], etc. In engineering practice, stone-matrix asphalt, with a 13-mm nominal maximum aggregate size (SMA-13) asphalt mixture [11], is a type of skeleton dense graded mixture and is now widely used in many roads that require better road performance to make driving safer and smoother. The oil–aggregate ratio of SMA mixtures are often relatively higher [12] than those of normal mixtures. Therefore, lignin fiber is now widely used in SMA-13 asphalt mixtures to absorb the asphalt in the mixtures [13,14,15]. Anti-rut agents are a kind of polymer additive used in road construction to enhance the properties of asphalt mixtures. For instance, ARA could help to increase the ductility and other properties of asphalt mortar [16] and the rutting resistance of asphalt mixtures [17]. ARA could comprehensively enhance all the properties of the SMA-16 asphalt mixtures, such as high- and low-temperature performances [18]. Research about using ARA and polyethylene (PE) together in an asphalt mixture was also conducted, and a good weight content combination of ARA and PE (0.2% and 0.5%, respectively), which could have a very good enhancement effect on the mixture, was also suggested [19].

The IDEAL-CT test [20] is a method to reveal the anti-crack ability of asphalt mixtures. It has two stages, namely the crack formation stage and the crack propagation stage. This paper adopted this test to reflect the crack resistance of asphalt mixtures and chose two indexes, W_initial_ and CT_Index_, to represent the anti-crack ability of SMA-13 asphalt mixtures in terms of crack formation and the propagation stage. Therefore, this paper aims to use this test to study the crack resistance of SMA-13 asphalt mixtures and to explore the effects of ARA on the anti-crack ability of the mixtures. In addition, the aging of asphalt mixtures could also affect the crack resistance of asphalt mixtures. The crack formation process of the asphalt mixture could be hardened in the aging process because the aging would usually make the texture inside the mixtures harder, which could cause it to have a tendency towards forming initial cracks, especially in roads where the volume of vehicles is very high. In the aging process, the basic change of the asphalt materials is mainly a change in chemical properties. There are several studies that have been conducted about the aging of asphalt mixtures [21,22] and the aging process was simulated using the indoor test proposed by AASHTO R30 [23].

There are already many researchers studying the anti-crack ability of pavement materials [24,25,26]; however, research mainly focuses on the physical property test. On the other hand, research the reasons for crack formation, or the strengthening mechanisms of the lignin fiber (LF), ARA, and other additives in asphalt mixtures are very limited. Therefore, this paper adopted FTIR spectroscopy measurements to reveal the interactions between LF and asphalt materials, and to reflect the effect of aging on asphalt materials in the lignin-fiber-reinforced asphalt mixtures, with and without ARA. FTIR spectroscopy measurements could be used to well reflect the chemical functional groups of the materials and this method also has some application in asphalt materials [27,28]. Thus, this paper tries to explore the effect of the aging process on the crack resistance of asphalt mixtures and the chemical properties of the asphalt material extracted from the test samples. The relationship between crack resistance and the parameters of the FTIR spectrum is studied to try to derive a prediction model for the crack resistance of asphalt mixtures using FTIR spectrum parameters.

Therefore, this paper adopted the IDEAL-CT test to analyze the effects of ARA on the crack performance of SMA-13 asphalt mixture. Asphalt material in the mixture was extracted using a rotary evaporation asphalt-recovery instrument. The FTIR spectrum was utilized to analyze the chemical properties of the asphalt inside the asphalt mixture. Grey correlation analysis [29] was adopted to select the most suitable FTIR parameters to predict the anti-crack ability of LFSMA-13, with and without ARA. The prediction model could also be used as a convenient method to test the crack resistance of LFSMA-13 asphalt mixtures in roads. This paper has certain significance in the prediction of the crack resistance of LFSMA-13 asphalt mixture, research concerning the strengthening mechanisms of ARA in LFSMA-13, and the study of the influence of aging degree on the crack resistance of LFSMA-13, with and without polymer-based ARA.

## 2. Materials and Gradation Design

### 2.1. Materials

#### 2.1.1. Asphalt

The styrene butadiene styrene (SBS)-modified asphalt utilized in this research was provided by Tiannuo Road Materials Technology Co., Ltd., Zhenjiang, China. The properties of the asphalt from the manufacturer, such as penetration, softening point, etc., are listed in Table 1. The test was conducted according to standards in China (JTG E20-2011).

#### 2.1.2. Lignin Fiber

**Figure 1:**
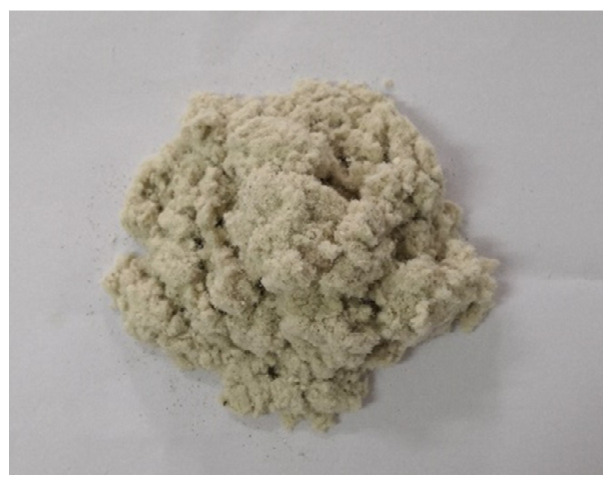
Photo of lignin fiber.

Lignin fiber (Figure 1) was used in this paper to modify the SMA-13 asphalt mixture because it is very commonly used in mixtures to absorb free asphalt inside a mixture in engineering practice. Therefore, this paper adopts lignin fiber produced by the JRS company, Rosenberg, Germany, to prepare lignin fiber (LF)-reinforced SMA-13. The properties of LF are shown in Table 2.

The texture of LF is very soft and it can easily curl with itself in a mixture [30]. The pH index of LF is greater than 7; therefore, LF is a type of alkaline material and it can interact well with asphalt materials because it is a kind of weak acid material [31]. The curled lignin fibers could strengthen the asphalt material because it can form a three-dimensional network structure inside the mixture.

#### 2.1.3. Anti-Rut Agent

**Figure 2:**
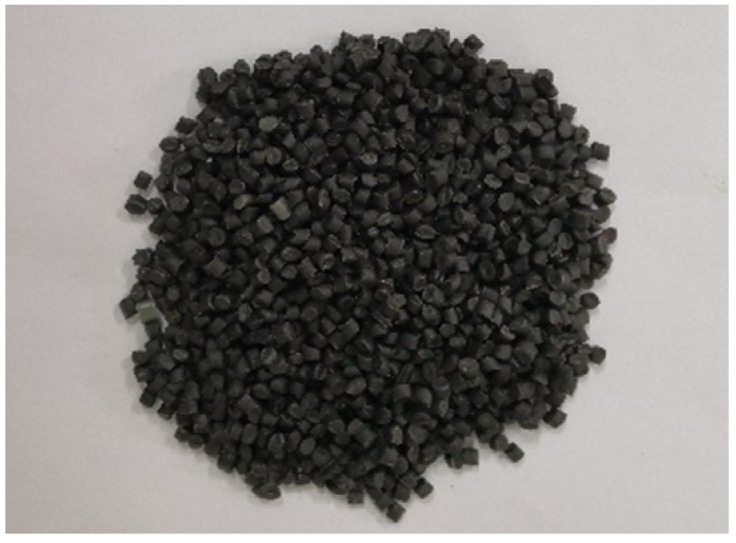
Anti-rut agent.

The polymer-based anti-rut agent (Figure 2) was made by Faon Transportation Technology (Shanghai, China) Co., Ltd. It is a kind of polymer additive used in asphalt materials to reinforce their performance. The property indexes are listed in Table 3. The melting point of ARA is lower than the mixing temperature of the asphalt mixture; therefore, ARA could melt inside the asphalt mixtures and form a network structure. The melted ARA could act as a bridge to connect the asphalt, mineral powder, or the aggregate together.

### 2.2. Gradation Design of SMA-13 Asphalt Mixtures with LF and ARA

**Figure 3:**
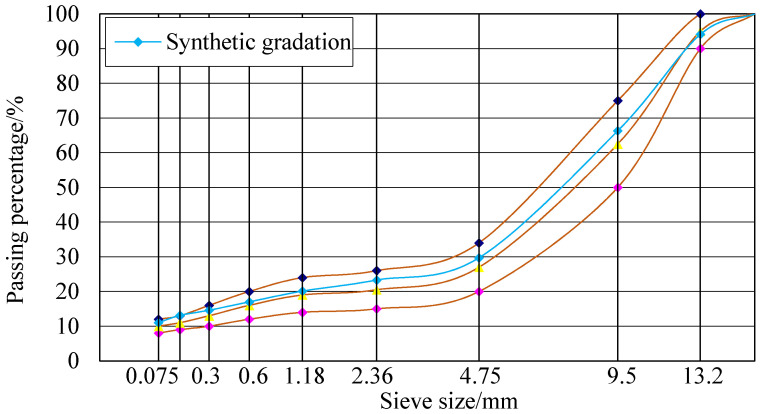
Gradation curve of SMA-13 asphalt mixture.

The gradation design curve of the mixture is shown in Figure 3 and the design results are shown in Table 4. The gradation design process was conducted following the procedures listed in the JTG F40-2004 standard in China [32]. The weight proportion of LF was set as 0.3%, which was suggested by previous research [33,34,35]. The manufacturer of ARA suggests that the ARA weight content be 0.3%–0.6%; thus, the weight proportion of ARA used in this study was set to 0.5% to make ARA-reinforced LFSMA-13 (ALSMA-13).

### 2.3. Sample Preparation

The short- and long-term aged LFSMA-13 and ALSMA-13 were prepared following the steps provided by the AASHTO standard testing method. The short-term aged mixtures were used to simulate asphalt mixtures after being paved and the long-term aged mixtures were prepared to simulate asphalt mixtures that have been paved for five to seven years. In the short-term aging process, the aggregates and asphalt were mixed and paved evenly on a plate and put into an oven for 4 h at 135 °C (under forced ventilation), and the loose mixture in the plate needed to be stirred once an hour in this process. The loose mixture was used to make the short-term asphalt mixture. After going through the aging process, the aged mixture was then put into an oven for 120 h at 85 °C (under forced ventilation) to prepare the long-term asphalt mixture samples.

## 3. Test and Analysis Methods

### 3.1. Test Methods

#### 3.1.1. IDEAL-CT Test

**Figure 4:**
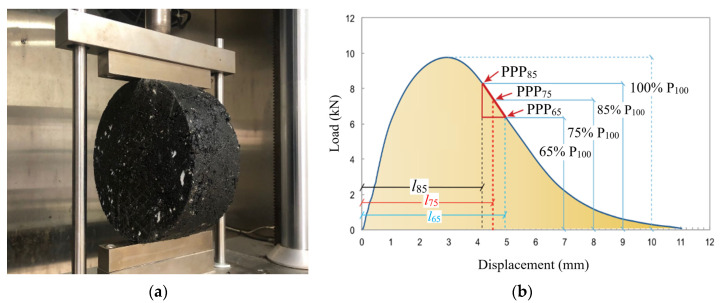
IDEAL test: (**a**) testing picture and (**b**) testing curve.

The test method to test the crack resistance of the asphalt mixtures adopted in this paper was the IDEAL-CT test and it has two test stages, a crack formation stage and a crack propagation stage. A test sample picture is shown in Figure 4a and the displacement–load curve is shown in Figure 4b. In this paper, the integral area of the displacement–load curve in the crack formation process is marked as W_initial_ (crack formation work) and it could reveal the work needed in the crack formation stage. After the crack was fully developed (the load reached the maximum point, P_100_), the most important part was the crack propagation rate. The crack propagation rate is represented by CT_Index_ and is calculated according to Equation (1). When CT_Index_ is bigger, the crack propagation rate is slower. In this equation, *G*_f_ is the fracture energy in the whole testing process and it is calculated using Equation (2). The *W*_f_ index in Equation (2) is the integral area of the displacement–load curve in the whole testing process. D is the specimen diameter (150 mm) and t is the specimen thickness (62 mm). |m_75_| is the slope of the displacement–load curve in the crack propagation process when the load is 75% that of the maximum load, P_100_, and l_75_ is the displacement at that point (PPP_75_). The definitions of PPP_85_ and PPP_65_ are similar to that of PPP_75_. |m_75_| is calculated according to these three points. The temperature in the testing process was set to 25 °C. All the tests were conducted three times (three samples tested for each composition) and the test was re-conducted if the error between any two test results was bigger than 5%. The average values of different indexes were adopted in this study. The load applied in the IDEAL test was not fixed. In the testing process, the loader kept moving downward at a rate of 50 mm/min. The machine used in this test was a Universal Testing Machine-25 (UTM-25) made by IPC Global, Melbourne, Australia.

(1) $$CT_{\mathrm{Index}}=\frac{G_{f}}{\left|m_{75}\right|}\times \frac{l_{75}}{D}$$

(2) $$G_{f}=\frac{W_{f}}{D\times t}\times 10^{6}$$

#### 3.1.2. FTIR Spectroscopy Measurements

**Figure 5:**
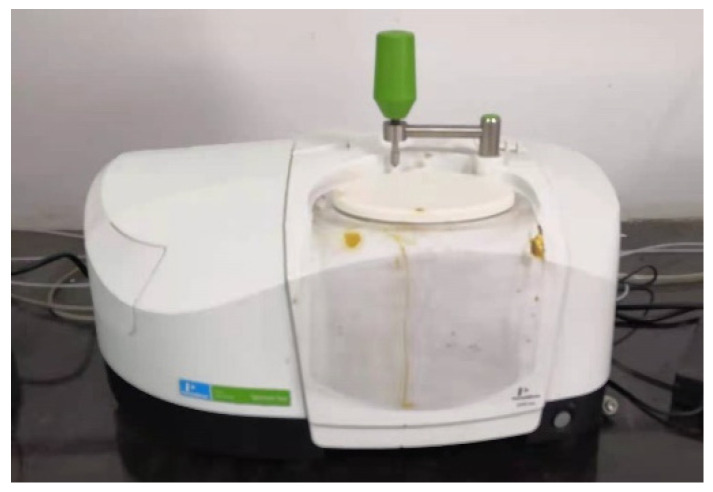
Fourier transform infrared spectrometer.

**Figure 6:**
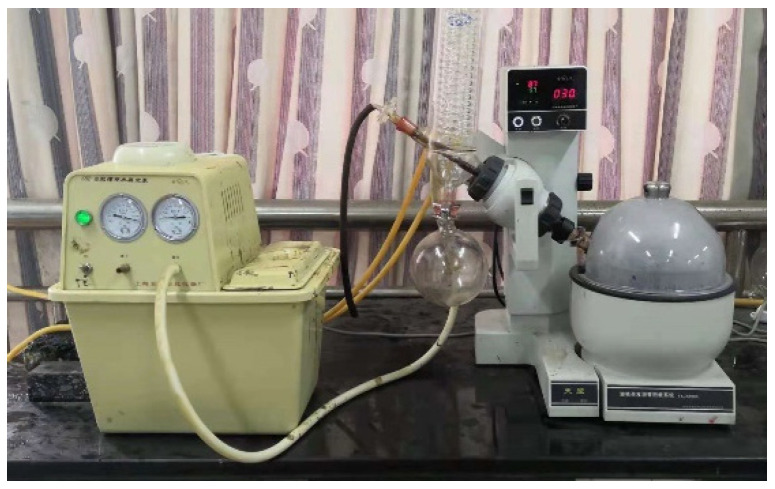
Rotary evaporation asphalt recovery instrument.

FTIR spectroscopy measurements were adopted to investigate the chemical composition of the asphalt extracted from LFSMA-13 and ALSMA-13 under different degrees of aging. Each sample was scanned 32 times in one single test to get the FTIR spectrum. The FTIR spectrometer was made by Perkinelmer Instruments Co., Ltd., Wellesley, MA, USA. The asphalt in the mixtures was extracted using a rotary evaporation asphalt-recovery instrument. The Fourier transform infrared spectrometer (Perkinelmer Instruments Co., Ltd., Wellesley, MA, USA) and the rotary evaporation asphalt-recovery instrument (Nanjing Tiankun Civil Engineering Materials Co., Ltd., Nanjing, China) are shown in Figure 5 and Figure 6 respectively. The peak areas in the FTIR spectrum of the asphalt in the mixtures were analyzed using OMNIC32 software after collecting the data of the peak areas.

#### 3.1.3. SEM Observations

SEM observations of the lignin fiber, anti-rut agent, and the distribution of anti-rut agent in the lignin fiber-reinforced asphalt mixtures were adopted in this study. The strengthening mechanisms of LF and ARA in the mixtures were also analyzed according to the micro images. The tests were conducted using a SEM made by Carl Zeiss Microscopy GmbH, Oberkochen, Germany. Test samples of the lignin fiber and ARA were pasted on an observation plate. The test samples of the asphalt mixtures were firstly frozen in a freezer and then were cut and pasted onto the observation plate. All test samples were being plated using gold powder with a vacuum coating machine. The test samples were then put into an observation chamber for observation.

### 3.2. Grey Correlation Analysis

Grey correlation analysis [29] is a method to get the order of certain influencing factors on a target index. A correlation degree value can be obtained after a series of calculations. When the correlation degree is bigger, the correlation of the corresponding influence factor with the target is bigger. First, data from the target (W_initial_ or CT_Index_) were listed in vector X_0_ and the data of the influence factors (peak areas of different peaks in the FTIR spectrum) were listed in vector X_*i*_; *i* is the number of the influencing factors. The specific data were illustrated in the analysis section. Target vector X_0_ was set as the reference vector. Second, all the data in the vectors were normalized using the first data point collected in the corresponding vector. Third, the absolute values of the difference in the corresponding data in the influence factor vectors and the target vector were collected in a new vector called |X_0_ − X_*i*_|. Then, the correlation degree between the target index and the influencing factors was calculated using Equation (5). The average correlation degree was calculated using Equation (6). In these equations, *k* is the number of the data points in a certain vector and m is the total number of data points in a vector. The correlation degree was marked as *ξ*. Equations (3) and (4) are the calculation equations for the minimum and maximum indexes of the vector of |X_0_ − X_*i*_| and are used in Equation (5). The ρ index in Equation (5) is the resolution coefficient and it is usually set as 0.5 [29].

(3) $$\left|\overset{n}{\underset{i=1}{\min}}\overset{m}{\underset{k=1}{\min}}\left|x_{0}(k)-x_{i}(k)\right|\right|$$

(4) $$\left|\overset{n}{\underset{i=1}{\max}}\overset{m}{\underset{k=1}{\max}}\left|x_{0}(k)-x_{i}(k)\right|\right|$$

(5) $$\xi (\left|x_{0}(k)-x_{i}(k)\right|)=\frac{\left|\overset{n}{\underset{i=1}{\min}}\overset{m}{\underset{k=1}{\min}}\left|x_{0}(k)-x_{i}(k)\right|\right|+\rho \times \left|\overset{n}{\underset{i=1}{\max}}\overset{m}{\underset{k=1}{\max}}\left|x_{0}(k)-x_{i}(k)\right|\right|}{\left|x_{0}(k)-x_{i}(k)\right|+\rho \times \left|\overset{n}{\underset{i=1}{\max}}\overset{m}{\underset{k=1}{\max}}\left|x_{0}(k)-x_{i}(k)\right|\right|}$$

(6) $$r=\frac{1}{m}\sum_{k=1}^{m}\xi_{k}$$

## 4. Results and Discussion

### 4.1. IDEAL-CT Test

**Figure 7:**
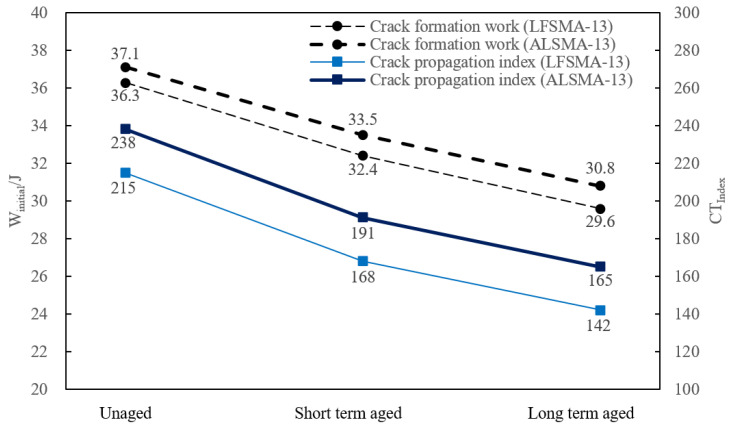
IDEAL-CT test result.

It could be derived from Figure 7 that, when the asphalt mixtures went through the short-term and long-term aging processes, the crack formation work, W_initial_, and crack propagation index, CT_Index_, decreased and the extent of the decrease was positively related with the degree of aging. It could be calculated that the W_initial_ of LFSMA-13 in the short-term and long-term situation decreased by 10.74% and 18.46%, respectively, compared with that of the unaged sample. The corresponding data of ALSMA-13 are 9.70% and 16.98%. In the meantime, the CT_Index_ of LFSMA-13 in the short-term and long-term situation decreased by 21.86% and 33.95% in comparison with the CT_Index_ of the unaged testing samples. The corresponding value of the CT_Index_ of ALSMA-13 were 19.75% and 30.67%, respectively. Generally, the aging of the test samples caused crack formation and the crack propagation index decreased; however, the decreasing extent of ALSMA-13 was a little bit lower than that of the LFSMA-13. It should also be mentioned that, even though the sensitivity of ALSMA-13 to aging was slightly lower than that of LFSMA-13, the crack formation work, W_initial_, and the crack propagation index, CT_Index_, of ALSMA-13 was all larger than that of LFSMA-13. It could be concluded that, in the unaged, short-term, and long-term situations, the W_initial_ values of ALSMA-13 were 2.20%, 3.40%, and 4.1% greater than that of LFSMA-13. Additionally, in the unaged situation, the CT_Index_ of ALSMA-13 was 10.70% larger than that of LFSMA-13; the corresponding data in the short-term and long-term conditions were 13.69% and 16.20%. Thus, it could be inferred from the test results that the difference between the crack resistance of LFSMA-13 and ALSMA-13 would be bigger when the aging degree increases.

### 4.2. FTIR Spectroscopy Measurements

**Figure 8:**
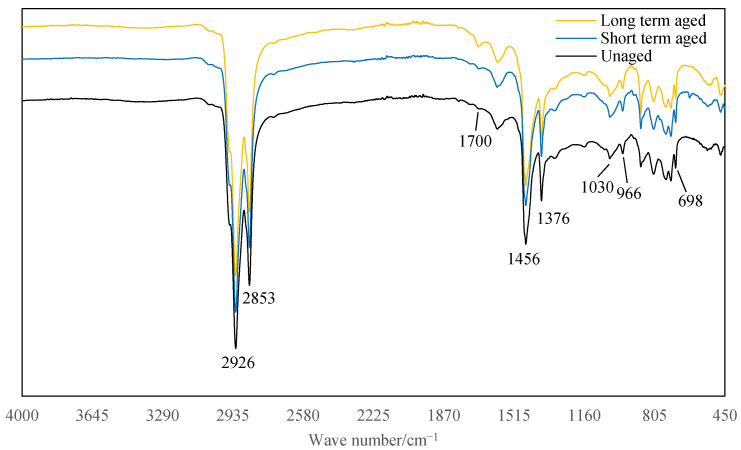
FTIR spectrum of asphalt extracted from LFSMA-13.

**Figure 9:**
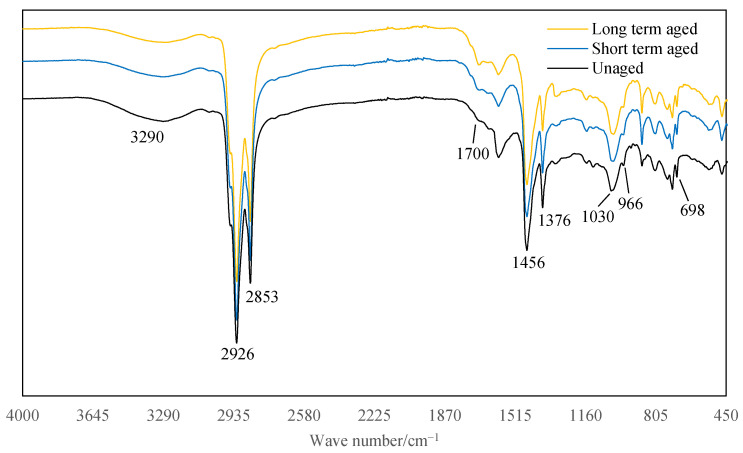
FTIR spectrum of asphalt extracted from ALSMA-13.

Figure 8 and Figure 9 show that the main difference between the FTIR spectra of asphalt extracted from LFSMA-13 and ALSMA-13 is the peak at 3290 cm^−1^, which is the characteristic peak of the imino group (–NH–) [36]. Therefore, the peak at 3290 cm^−1^ is caused by the anti-rut agent because the difference between the LFSMA-13 and ALSMA-13 materials was the anti-rut agent. It could be concluded from the FTIR spectrum of the traditional SBS modified asphalt [37], that the spectrum of asphalt extracted from LFSMA-13 is similar to that of the SBS-modified asphalt. Therefore, ARA could chemically interact with the SBS-modified asphalt and LF only physically interacted with SBS-modified asphalt.

The peaks in the spectrum of the asphalt material extracted from LFSMA-13 are at 2926 cm^−1^, 2853 cm^−1^, 1700 cm^−1^, 1456 cm^−1^, 1376 cm^−1^, 1030 cm^−1^, 966 cm^−1^, and 698 cm^−1^. The peaks at 2926 cm^−1^ and 2853 cm^−1^ are caused by the antisymmetric and symmetric stretching vibration of methylene (–CH_2_–) [38] and the peaks at 1456 cm^−1^ and 1376 cm^−1^ are caused by the vibration of methyl (–CH_3_–) [39,40]. The peaks at 1700 cm^−1^ and 1030 cm^−1^ are caused by the carbonyl [41] and sulfoxide group [42], respectively, and these two peaks could usually be adopted to represent the aging degree of asphalt materials. The peaks at 966 cm^−1^ and 698 cm^−1^ are caused by the SBS modifier in the SBS-modified asphalt [37] and are caused by the trans butadiene and styrene.

The peak areas listed above are listed in Table 5; the peak areas could be utilized to represented the content of the related chemical functional groups. It can be seen from Table 5 that, when the aging degree increases, the peak area of the imino group (3290 cm^−1^) in the spectrum of the asphalt extracted from ALSMA-13 decreases; the peak areas at 2926 cm^−1^ and 2853 cm^−1^ (peaks of methylene) of all the samples have some small fluctuations but are relatively stable, the peak areas of the carbonyl (1700 cm^−1^) and sulfoxide groups (1030 cm^−1^), which represent the aging degree of asphalt, increase. The peak areas at 1456 cm^−1^ (bending vibration of methyl) increase and the peak areas at 1376 cm^−1^ (scissoring vibration of methyl) decrease; and the peak areas of trans butadiene (966 cm^−1^) and styrene (698 cm^−1^) in the samples decrease.

This study adopted three kinds of aging degrees, therefore, each target vector (the W_initial_ and CT_Index_ vectors) had three data points. Thus, three kinds of influence factors (three kinds of peak areas) are needed to precisely predict the W_initial_ and CT_Index_ by solving the ternary linear equations. There are many peaks listed above, thus, in order to select the most suitable three areas to derive the linear prediction model of the W_initial_ and CT_Index_, grey correlation analysis was adopted to choose the most suitable peak areas (the peak areas were chosen when the correlation degree ranked in the top three). It should also be mentioned that, this paper, the three aging degrees suggested by the Strategic Highway Research Program (SHRP) were selected to get the asphalt mixtures with different aging degrees; therefore, the linear prediction model could be precisely derived using three kinds of peak areas. In the future, more aging times or aging degrees could be adopted to get a more precise prediction model.

### 4.3. Grey Correlation Analysis

The procedures of conducting grey correlation analyses are listed in the section above. Therefore, this section takes the grey correlation analysis result between the CT_Index_ (the target vector) of LFSMA-13 and the peak areas of the selected peaks (the influence factor vector) of the FTIR spectrum of asphalt extracted from LFSMA-13 as an example to show the process of grey correlation analysis. The related grey correlation data between CT_Index_ (the data are listed in Figure 8) and different peak areas are listed in Table 6, and the normalized index of the data are listed in Table 7. The data on the absolute value of the difference between X_0_ and X_i_ are listed in Table 8. Finally, the correlation degree data are listed in Table 9. After conducting similar analyses, the grey correlation analysis results between the W_initial_ of LFSMA-13 and the peak areas of the selected peaks of the FTIR spectrum of asphalt extracted from LFSMA-13 are listed in Table 10. The grey correlation analysis results between the CT_Index_ and W_initial_ of ALSMA-13 and the different peak areas are listed in Table 11 and Table 12, respectively. After conducting grey correlation analyses, the peak areas chosen to derive the linear prediction model of the CT_Index_ of LFSMA-13 are at 1376 cm^−1^, 966 cm^−1^ and 698 cm^−1^. The chosen peak areas to predict the CT_Index_ of ALSMA-13 are at 3290 cm^−1^,1376 cm^−1^ and 966 cm^−1^. The peak areas selected to predict the W_initial_ of LFSMA-13 and ALSMA-13 are at 1376 cm^−1^, 966 cm^−1^ and 698 cm^−1^. The ternary linear prediction models are shown in the next section.

### 4.4. Preliminary Prediction Model and Application Method

The peak areas chosen to derive the preliminary prediction model of the W_initial_ and CT_Index_ of LFSMA-13 and ALSMA-13 are listed in the section above. Therefore, this section lists the results of the ternary linear prediction models of W_initial_ and CT_Index_ of LFSMA-13 and ALSMA-13. The peak areas at 3290 cm^−1^, 1376 cm^−1^, 966 cm^−1^ and 698 cm^−1^ are marked as PA_3290_, PA_1376_, PA_966_ and PA_698_. In addition, the ternary linear prediction models of the W_initial_ and CT_Index_ of LFSMA-13 are listed in Equations (7) and (8). The ternary linear prediction models of the W_initial_ and CT_Index_ of ALSMA-13 are listed in Equations (9) and (10). These preliminary prediction models could be modified in the future when more aging degrees are adopted to get the more precise prediction models of the crack resistance of LFSMA-13, with and without anti-rut agent. The models in this section are just the preliminary prediction models of the crack resistance of LFSMA-13 and ALSMA-13 and could be utilized to roughly predict crack resistance.

(7) $$CT_{\mathrm{Index}}=1.411\times PA_{1376}+8.365\times PA_{966}-11.218\times PA_{698\;\;}(\mathrm{LFSMA}-13)$$

(8) $$W_{\mathrm{initial}}=0.165\times PA_{1376}+0.335\times PA_{966}+0.031\times PA_{698\;\;}(\mathrm{LFSMA}-13)$$

(9) $$CT_{\mathrm{Index}}=0.186\times PA_{3290}+1.349\times PA_{1376}-6.686\times PA_{966\;\;}(\mathrm{ALSMA}-13)$$

(10) $$W_{\mathrm{initial}}=0.025\times PA_{1376}+1.409\times PA_{966}+0.553\times PA_{698\;\;}(\mathrm{ALSMA}-13)$$

**Figure 10:**
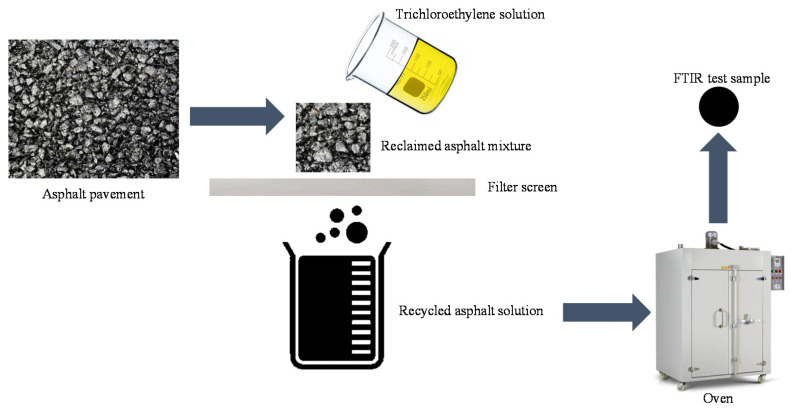
Method to get the FTIR spectroscopy measurement sample.

They could also be seen as a convenient method to quickly test the crack resistance of LFSMA-13, with and without anti-rut agent, in engineering practice by measuring the FTIR spectrum of asphalt materials extracted from mixtures. This section also provides a quick method to determine the asphalt material in mixtures, needed for FTIR spectroscopy measurements. The method to quickly determine the test sample of the asphalt materials in an asphalt mixture is illustrated in Figure 10. First, the reclaimed asphalt mixture was collected from the asphalt pavement. Second, a trichloroethylene solution was used to wash asphalt material from the surface of the aggregates, after which, it was being filtered using the filter screen, which allows the recycled asphalt solution to pass through while screening the mineral powder. The asphalt material sample needed for FTIR spectroscopy measurements is very limited; therefore, the content of the recycled asphalt solution was also very small. Thus, it could be put into a small oven with a special container to collect the trichloroethylene vapor to acquire the FTIR spectroscopy measurement sample (at 87.1 °C, the boiling point of trichloroethylene). All the processes could be conducted very quickly, because the content of the sample needed for FTIR spectroscopy measurements is very small. After conducting the FTIR spectroscopy measurements, the selected peak areas could be collected and the crack resistance of the asphalt mixtures could be predicted. The aging degree could also be derived by finding the corresponding crack resistance test index in the test curve of the crack resistance test, such as in Figure 8. In the future, more aging degrees could be utilized to derive a more precise model to predict crack resistance and infer the aging degree of asphalt mixtures of a road.

### 4.5. SEM Observations Result

**Figure 11:**
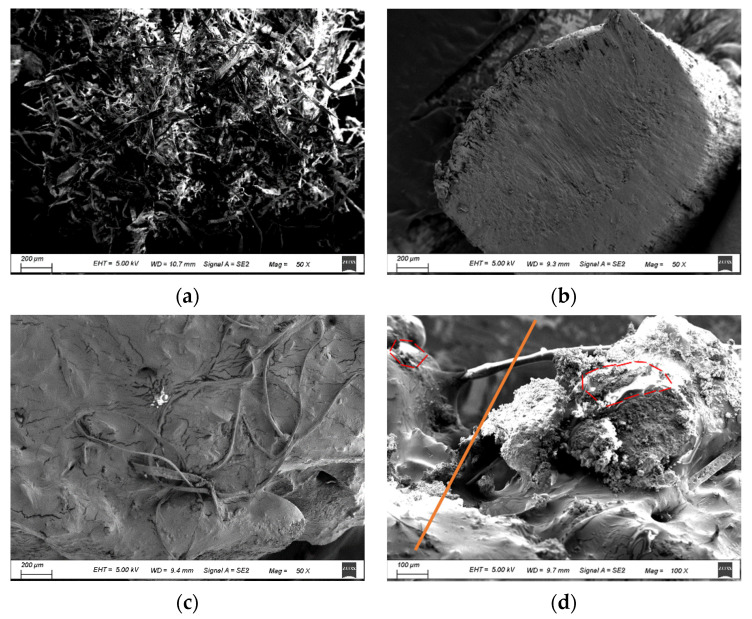
SEM observations result: (**a**) lignin fiber (50×); (**b**) anti-rut agent (50×); (**c**) LF-reinforced mixture with ARA (50×); (**d**) LF-reinforced mixture with ARA (100×).

Figure 11 shows the micro image of LF, ARA, and the LF-reinforced asphalt mixture with ARA. Micrographs of LF showed that LF could curl easily. The surface of ARA is not very smooth and it has some tiny bulges on its surface. By comparing the micro-morphology of the fibers in Figure 11a,c, the surface of the lignin fibers were found to be surrounded by the asphalt film and then it could act as a bridge to connect the aggregate and asphalt, making the asphalt composite material more solid. It could be seen from Figure 11c that the ARA could melt into the asphalt during the mixing process, which could help to make the ARA participate more in bearing the stress inside the mixtures. The aging process mainly affects the properties of the asphalt and the ARA inside the mixture. Therefore, it is reasonable to use the chemical composition index extracted from the asphalt mixtures to reflect the change in properties, because the fundamental change of the materials caused by the aging is related with the chemical composition. In the aging process, the chemical functional groups in ARA or the asphalt could change, which could result in a change in the physical properties of asphalt or ARA, etc., in the mixtures. The change in the physical properties of asphalt or ARA will affect the physical properties of asphalt mixtures. For instance, it could be seen from the preliminary prediction equation of the CT_Index_ of ALSMA-13 (Equation (9)), after adding ARA into LFSMA-13, the peak areas at 3290 cm^−1^ (the special peak in ARA) was utilized to reveal the CT_Index_ (the index related to the crack resistance in the crack propagation process). However, it can also be seen that all the preliminary prediction equations (Equations (7)–(10)) showed that, in the crack formation stage, the main factor affecting crack resistance indexes are the FTIR spectrum parameters of the asphalt material in the mixtures. This is mainly because, in the crack formation stage, the asphalt material, ARA, and aggregates are stuck together (as shown in Figure 11c) and the asphalt content is much greater than that of ARA. Therefore, it is reasonable that the chemical functional groups of the asphalt materials are the most important ones in the crack formation stage for LFSMA-13 and ALSMA-13. Additionally, in the crack formation stage, after adding ARA into LFSMA-13, as shown in Figure 11d, the ARA could act as a bridge to connect the asphalt binders on the two sides of the micro cracks, which could slow the crack propagation rate. The red circle in Figure 11d is the melted ARA and the orange line shows the direction of the cracks. Since the peak areas of certain peaks could well reflect the content of certain chemical functional groups in the asphalt materials and ARA, it is quick to analyze the change in the crack resistance of the asphalt mixture using the FTIR spectroscopy measurement analysis and the derived preliminary prediction models of the crack resistance test indexes.

## 5. Conclusions

After conducting tests of the crack resistance of LFSMA-13, with and without ARA, the chemical composition index of the asphalt material extracted from the test samples, the grey correlation analyses, and the prediction model analyses, the conclusions listed below could be drawn:

(1) The sensitivity of ALSMA-13 to aging is slightly lower than that of LFSMA-13. The crack formation work, W_initial_, and the crack propagation index, CT_Index_, of ALSMA-13 are higher than those of LFSMA-13. The difference between the crack resistances of LFSMA-13 and ALSMA-13 will be higher when the aging degree increases.
(2) When the aging degree increase, the peak area of the imino group in ALSMA-13 decreases, the peak areas of methylene of all the samples are relatively stable, the peak areas of the carbonyl and sulfoxide groups increase, and the peak areas of trans butadiene and styrene in the samples decrease.
(3) Grey correlation analysis could be adopted to select suitable indexes of the FTIR spectra to derive prediction models of CT_Index_ and W_initial_ of LFSMA-13 and ALSMA-13. The ternary linear prediction models could also well predict the crack resistance.
(4) A convenient method or procedure to quickly test the crack resistance of the LFSMA-13, with and without anti-rut agent, was provided in the research. SEM images showed that LF curled in the mixtures and ARA melted in the asphalt.

## Figures and Tables

**Table 1:** Test results of performance of the SBS-modified asphalt.

Index	Value
Penetration (25 °C, 100 g, 5 s), 0.1 mm	71
Softening point, °C	64
Ductility (5 °C, 5 cm/min), cm	48
PI index	0.5
Solubility, %	99.9
Elastic recovery (25 °C), %	76
Rotational viscosity (135 °C), Pa·s	2.302
Relative density (25 °C)	1.031

**Table 2:** Properties of lignin fiber.

Index	Lignin Fiber
Specific surface area, m^2^/g	1.93
Aspect ratio	100
Hygroscopic rate, %	28.70
Heat resistance, °C	260
Ph value	7.6
Fracture strength, MPa	<300

**Table 3:** Properties of the anti-rut agent.

Index	Anti-Rut Agent
Density, g/cm^3^	0.96
Diameter, mm	2–3
Melting point, °C	135–150
Polymer content, %	≥95

**Table 4:** Gradation design results of SMA-13.

Index	Optimum Asphalt Content (OAC)/%	Air Voids (VV)/%	Voids in Mineral Aggregate (VMA)/%	Voids Filled with Asphalt (VFA)/%	Stability/kN	Flow Value/mm
LFSMA-13	6.0	3.8	17.2	78.0	11.93	2.8
ALSMA-13	6.0	3.9	17.3	77.5	14.38	2.6
Specification requirements	/	3~4.5	≥16.5	70~85	≥6	2.0~4.0

**Table 5:** Characteristic peak areas of asphalt material extracted from mixtures.

Aging Degree	Mixture	3290 cm^−1^	2926 cm^−1^	2853 cm^−1^	1700 cm^−1^	1456 cm^−1^	1376 cm^−1^	1030 cm^−1^	966 cm^−1^	698 cm^−1^
Peak area (unaged)	ALSMA-13	814.043	1301.605	399.881	9.118	739.81	140.834	204.736	15.495	21.255
	LFSMA-13	/	1370.071	415.502	6.236	754.708	145.334	129.157	34.395	24.767
Peak area (short term)	ALSMA-13	558.818	1344.04	422.75	21.106	865.316	131.556	236.645	13.543	20.138
	LFSMA-13	/	1384.12	422.454	7.997	837.843	134.164	167.747	28.416	23.093
Peak area (long term)	ALSMA-13	511.145	1337.038	414.275	46.824	848.767	111.202	254.31	11.997	20.114
	LFSMA-13	/	1361.256	413.794	26.378	859.53	119.484	184.64	27.330	22.754

**Table 6:** Grey correlation data between CT_Index_ and different peak areas (LFSMA-13).

Aging Degree	Unaged	Short Term	Long Term
CT_Index_ (X_0_)	215	168	142
Peak area at 2926 cm^−1^ (X_1_)	1370.071	1384.120	1361.256
Peak area at 2853 cm^−1^ (X_2_)	415.502	422.454	413.794
Peak area at 1700 cm^−1^ (X_3_)	6.236	7.997	26.378
Peak area at 1456 cm^−1^ (X_4_)	754.708	837.843	859.530
Peak area at 1376 cm^−1^ (X_5_)	145.334	134.164	119.484
Peak area at 1030 cm^−1^ (X_6_)	129.157	167.747	184.640
Peak area at 966 cm^−1^ (X_7_)	34.395	28.416	27.330
Peak area at 698 cm^−1^ (X_8_)	24.767	23.093	22.754

**Table 7:** Normalized index of the grey correlation data between CT_Index_ and different peak areas (LFSMA-13).

Aging Degree	Unaged	Short Term	Long Term
CT_Index_ (X_0_)	1	0.781395	0.660465
Peak area at 2926 cm^−1^ (X_1_)	1	1.010254	0.993566
Peak area at 2853 cm^−1^ (X_2_)	1	1.016732	0.995889
Peak area at 1700 cm^−1^ (X_3_)	1	1.282393	4.229955
Peak area at 1456 cm^−1^ (X_4_)	1	1.110155	1.138891
Peak area at 1376 cm^−1^ (X_5_)	1	0.923143	0.822134
Peak area at 1030 cm^−1^ (X_6_)	1	1.298784	1.429578
Peak area at 966 cm^−1^ (X_7_)	1	0.826167	0.794592
Peak area at 698 cm^−1^ (X_8_)	1	0.932410	0.918722

**Table 8:** Calculation of the absolute value of the difference between X_0_ and X_i_.

Aging Degree	Unaged	Short Term	Long Term
X_1_ − X_0_	0	0.228859	0.333101
X_2_ − X_0_	0	0.235336	0.335424
X_3_ − X_0_	0	0.500997	3.569490
X_4_ − X_0_	0	0.328760	0.478426
X_5_ − X_0_	0	0.141747	0.161669
X_6_ − X_0_	0	0.517388	0.769113
X_7_ − X_0_	0	0.044771	0.134127
X_8_ − X_0_	0	0.151015	0.258257

**Table 9:** Grey correlation degree between CT_Index_ and different peak areas (LFSMA-13).

Aging Degree	Unaged	Short Term	Long Term	Average Value
Peak area at 2926 cm^−1^	1	0.886344	0.842717	0.909687
Peak area at 2853 cm^−1^	1	0.883502	0.841794	0.908432
Peak area at 1700 cm^−1^	1	0.780816	0.333333	0.704717
Peak area at 1456 cm^−1^	1	0.844448	0.788604	0.877684
Peak area at 1376 cm^−1^	1	0.926422	0.91694	0.947787
Peak area at 1030 cm^−1^	1	0.775257	0.698843	0.824700
Peak area at 966 cm^−1^	1	0.975528	0.930101	0.968543
Peak area at 698 cm^−1^	1	0.921987	0.873589	0.931859

**Table 10:** Grey correlation degree between W_initial_ and different peak areas (LFSMA-13).

Aging Degree	Unaged	Short Term	Long Term	Average Value
Peak area at 2926 cm^−1^	1	0.93551	0.905517	0.947009
Peak area at 2853 cm^−1^	1	0.932201	0.904402	0.945534
Peak area at 1700 cm^−1^	1	0.814109	0.333333	0.715814
Peak area at 1456 cm^−1^	1	0.886956	0.840715	0.909224
Peak area at 1376 cm^−1^	1	0.982403	0.996087	0.99283
Peak area at 1030 cm^−1^	1	0.807795	0.735441	0.847746
Peak area at 966 cm^−1^	1	0.962566	0.987944	0.983503
Peak area at 698 cm^−1^	1	0.977192	0.942948	0.97338

**Table 11:** Grey correlation degree between CT_Index_ and different peak areas (ALSMA-13).

Aging Degree	Unaged	Short Term	Long Term	Average Value
Peak area at 3290 cm^−1^	1	0.950345	0.97141	0.973918
Peak area at 2926 cm^−1^	1	0.906132	0.869296	0.925143
Peak area at 2853 cm^−1^	1	0.897133	0.866321	0.921151
Peak area at 1700 cm^−1^	1	0.594929	0.333333	0.642754
Peak area at 1456 cm^−1^	1	0.858152	0.830282	0.896145
Peak area at 1376 cm^−1^	1	0.944063	0.958436	0.967499
Peak area at 1030 cm^−1^	1	0.862749	0.801848	0.888199
Peak area at 966 cm^−1^	1	0.968811	0.964825	0.977879
Peak area at 698 cm^−1^	1	0.938745	0.897723	0.945489

**Table 12:** Grey correlation degree between W_initial_ and different peak areas (ALSMA-13).

Aging Degree	Unaged	Short Term	Long Term	Average Value
Peak area at 3290 cm^−1^	1	0.908617	0.914101	0.940906
Peak area at 2926 cm^−1^	1	0.943197	0.916142	0.953113
Peak area at 2853 cm^−1^	1	0.933143	0.912734	0.948626
Peak area at 1700 cm^−1^	1	0.603914	0.333333	0.645749
Peak area at 1456 cm^−1^	1	0.889767	0.871607	0.920458
Peak area at 1376 cm^−1^	1	0.985733	0.981491	0.989075
Peak area at 1030 cm^−1^	1	0.894869	0.839367	0.911412
Peak area at 966 cm^−1^	1	0.986733	0.974671	0.987135
Peak area at 698 cm^−1^	1	0.979754	0.948812	0.976189

## Data Availability

The data presented in this study are available on request from the corresponding author.
